# Effects of Sodium Cromoglycate on Iranian Asthmatic Subjects Without Exposure to any Bronchoconstrictor agent

**Published:** 2012

**Authors:** Tajmah Mombeini, Mohammad Reza Zahedpoure-Anaraki, Ahmad Reza Dehpour

**Affiliations:** a *Department of Pharmacology, School of Medicine, Shahed University, Tehran, Iran. *; b *Department of Pharmacology, School of Medicine, Shahid Beheshti University of Medical Sciences, Tehran, Iran.*; c *Department of Pharmacology, School of Medicine, Ahwaz -Jondishapour University of Medical Sciences (AJUMS ), Ahwaz, Iran.*; d *Department of Internal Medicine, Imam-Khomeini Hospital, School of Medicine, Tehran University of Medical Sciences, Tehran, Iran.*; e *Department of Pharmacology, School of Medicine, Tehran University of Medical Sciences, Tehran, Iran.*

**Keywords:** Cromolyn sodium, Lung function tests, Forced expiratory flow volume in one second, Bronchial asthma, Adults

## Abstract

Cromolyn sodium, a mast cell stabilizing agent, provides an immediate protective effect against the exercise-induced bronchoconstriction while being used before the exercise. However, cromolyn is ineffective in reversing asthmatic bronchospasm; it is used as a maintenance therapy and has a prophylactic role in chronic asthma.

The purpose of this study was to determine the extent of change in baseline lung function tests following a single dose of cromolyn sodium in adult asthmatics.

Forty volunteers (33 women and 7 men) with moderate to severe persistent asthma were randomly assigned to receive 20 mg cromolyn, 40 mg cromolyn or cromolyn-placebo. The percent of improvement in lung function parameters was compared among the groups, during 1 h of inhalation.

Low dose of cromolyn induced more improvement in most lung function parameters such as forced expiratory flow volume in one second, forced vital capacity and peak expiratory flow compared with other groups. After 15 min, the improvement percentage of baseline forced expiratory flow volume in one second was 3.35 ± 1.5, for sodium cromoglycate-20 mg group compared with 0.98 ± 1.43 and - 0.68 ± 1.2 for sodium cromoglycate-placebo and sodium cromoglycate-40 mg, groups respectively. However, the differences between means were not significant. Furthermore, based on the definition of American Thoracic Society (ATS) for a ”significant post-bronchodilator response” developed in a few patients 15 min after the inhalation of 20 mg cromolyn sodium.

It is suggested that probably the inhalation of 20 mg of cromolyn sodium could immediately improve the lung function in few adults with asthma.

## Introduction

Asthma is a common worldwide problem, with an estimated 300 million affected individuals. Asthma is a chronic inflammatory disorder of airways in which many cells and cellular elements play a role. To treat asthma, medications can be classified as the relievers or controllers. Relievers are medications used on an as-needed basis that act quickly to reverse the bronchoconstriction and relieve its symptoms. Controllers are medications taken daily on a long-term basis to keep asthma under clinical control chiefly through their anti-inflammatory effects (1). Sodium cromoglycate (SCG) and inhaled corticosteroids have become established as the effective controller medications and maintenance treatments for children and adults with asthma (2-6). Cromolyn has been reported to have a variety of activities that may relate to its therapeutic effect in asthma such as its effects on some cell types (especially mast cell and other leukocytes) and chemical mediators involved in chronic airway inflammation (7). Previously, SCG was used for the treatment of mild-to-moderate asthma, however, recently it has been reported that cromolyn alone or in combination with beta agonist salbutamol leads a good control of asthma in both children and adults with severe refractory and oral steroid-dependent asthma (8-11).

Sodium cromoglycate is also an effective drug for preventing exercise-induced asthma and asthma induced by other inhalational challenges such as mannitol (12-15). A single dose of drug can immediately prevent falling in forced expiratory flow volume in one second (FEV1) following the exercise in exercise-induced asthma. So, this question would come up whether it may also have any effect on baseline lung function in chronic asthma.

Previously, it was showed that cromolyn has no effect on the baseline FEV1 up to 30 min after the inhalation, before challenge by exercise or water in asthmatic subjects (16, 17). Although other lung function parameters are also important, such as forced vital capacity (FVC) and peak expiratory flow (PEF), however immediate effects of cromolyn on these parameters have not been evaluated in asthmatic subjects, previously (18). Also, the effect of pretreatment or treatment with cromolyn has not been evaluated in Iranian asthmatic subjects, previously.

So, the purpose of the present work is to determine the effect of a single dose of inhaled sodium cromoglycate on lung function variables in adults, with moderate to severe persistent asthma, during 1 h of inhalation.

## Experimental

*Patients*

Among patients with chronic asthma who referred to the Internal Medicine Department of Imam-Khomeini Hospital, forty volunteers with a documented diagnosis of bronchial asthma and reversible airflow obstruction with a FEV1 < 80% of predicted value, were entered to the study. According to the Global Initiative for Asthma (GINA) guidelines, the severity of their disease was classified as moderate persistent or severe persistent asthma, step 3 or 4, respectively (1). Volunteers included 33 women and 7 men with the age range of 27-67 years (mean ± SEM, 41 ± 1.69) whose characteristics are presented in Table 1. All were non-smoker, free of upper or lower respiratory tract infections within 1 month of study, according to the medical history. All volunteers were in a stable condition with respect to their asthma control, before the study. Beta-2 adrenoceptor agonists were withheld for 6 h before the study, methylxanthines and SCG were withheld for 24 h, and aerosol steroids were withheld on the morning of the study day (19). The research followed the tenets of the Declaration of Helsinki promulgated in 1964 and was approved by the Ethic Committee of Medical School and a written consent was obtained from each patient after that the explanation of the study was given.

**Table 1:** Demographic characteristics of patients (A, B, C).

**A: Sodium cromoglycate-placebo group (SCG-P)**						
**Patient No.**	**Gender**	**Age (y)**	**Height (cm)**	**Weight (kg)**	**% pred. FEV1**	**Current medications**
1	F	50	154	64	66.47	S
2	F	41	147	68	70.45	S
3	M	45	170	68.7	65.81	S, A
4	F	40	160	69	44.89	S
5	F	35	161	75.5	77.49	S
6	F	30	166	64.8	70.37	S
7	F	28	160	82.7	42.18	S
8	F	32	149	57	56.86	S, A
9	F	28	152	61.5	62.23	S, A, P
10	M	34	173	93	72.73	S
11	F	65	148	68	76.38	S, P, B, A
12	F	57	149	68	48.76	S, C, T
13	F	28	150	64.5	47.62	S, A
14	F	57	157	81	49.04	S, P
Mean (SEM)		40.7 (12.3)	156.9 (8.5)	70.4 (9.6)	60.8 (12.3)	
**B: Sodium cromoglycate 20-group (SCG-20)**						
**Patient No.**	**Gender**	**Age(y)**	**Height (cm)**	**Weight (kg)**	**% pred. FEV1**	**Current medications**
1	F	28	150	64.5	47.62	S, A
2	M	29	172	78	71.67	S
3	M	34	180	85.6	75.59	S
4	M	43	174	84.3	56.68	S
5	F	35	158	63.8	47.11	S
6	M	35	166	78	37.74	S, A
7	M	48	175	65.4	35.99	S, A, P, C
8	F	45	153	63.2	52.52	S
9	F	50	158	62	61.05	S, A, P
10	F	35	152	73	73.93	S
11	F	58	155	57	59.44	S
12	F	50	158	76	61.4	S
13	F	57	157	81	49.04	S, P
14	F	38	168	75	46.56	S, T, D
Mean (SEM)		41.8(9.8)	162.6 (9.7)	71.9 (9.1)	55.5 (12.5)	
**C: Sodium cromoglycate 40-group (SCG-40)**						
**Patient No.**	**Gender**	**Age(y)**	**Height (cm)**	**Weight (kg)**	**% pred. FEV1**	**Current medications**
1	F	27	166	72	67.36	S, A
2	F	34	154	82	59.87	S, P, C, T
3	F	45	164	78.5	68.4	S
4	F	32	151	56.4	67.75	S, P, T
5	F	47	159.5	78	69.97	S, C
6	F	40	168	85.5	75.91	S
7	F	36	152	72.2	52.67	S
8	F	34	152	60.7	71.24	S
9	F	34	165	81	63.26	S, A, P
10	F	41	157	61.5	71.34	S
11	F	67	164	70	64.12	S
12	F	48	159.5	75	79	S, T
Mean (SEM)		40.4 (10.1)	159.3 (5.8)	72.7 (8.8)	67.6 (6.7)	

A: Aminophylline, B: Beclomethasone inhaler, C:Cromolyn sodium, D:Dexamethasone, P: Prednisolone, S: Salbutamol inhaler T: Theophylline

*Study protocol*

The subjects attended the laboratory on two days: the 1^st^ day and 2 days later. In the first day, the subjects were instructed on how to perform lung function test (Pulmonary product, Fukuda Sangio, Model: FUDAC-50-3 Tokyo, Japan) and the technique of using drug through the spinhaler (Spinhaler, Turbo-Inhaler, Turbo-Inhalateur, Fisons Pharmaceuticals, Loughbrough, UK). In the second day, on arrival at the laboratory, the subjects rested for 15 min and then performed three lung function maneuvers; the best value was taken. Thereafter, patients were entered randomly to each study group; sodium cromoglycate-20 mg (SCG-20 group) (Intal^®^*,* Fisons Pharmaceuticals, Loughbrough, UK), sodium cromoglycate-40 mg (SCG-40 group) or cromolyn-placebo (SCG-P group, which patients inhaled an emptied and cleaned capsule of SCG). Two dosages of drug are used to determine if cromolyn has a dose-dependent effect in this setting. Patients must inhaled deep breaths of drug powder or placebo through a spinhaler, from residual volume (RV) to total lung capacity (TLC), with breath-holding for 10 sec (20). Then, lung function tests were repeated at 15, 30 and 60 min after the treatment.

*Data analysis*

Data were expressed as mean ± SEM. The effect of treatments on lung function variables including FEV_1_, FVC, PEF and maximal expiratory flow were studied when 75%, 50% and 25% of the forced vital capacity (MEF_75%_, MEF_50%_ and MEF_25%_ respectively) remained in the lung.

Changes from baseline values were calculated through the following formula:

% Improvement = (Predicted Value at That Time - Baseline Value) × 100 / Baseline Value

The normality of distribution was assessed using Kolmogorov-Smirnov test. Then, in case of normal distribution, the mean of the baseline values of lung function were compared through ANOVA and the means of improvement were compared through repeated measure ANOVA, between the SCG-P, SCG-20 and SCG-40 groups. A p-value of less than 0.05 was considered as statistically significant.

## Results and Discussion

According to the information obtained by history, most patients did not use antiasthma drugs regularly, and approximately their asthma has not been controlled appropriately (Table 1). Differences between the mean of baseline values of lung function parameters including the percentage predicted FEV_1_, FVC and PEF, were not statistically significant between the groups (Table 2). However, no treatment significantly changed related baseline values of lung function at 15, 30 or 60 min.

**Table 2:** Means of percent of predicted value of lung function parameters before and after treatments in the adult asthmatics

**`Parameter**	**Time**	**Placebo (SCG-P) (n = 14)**	**Cromolyn 20 mg (SCG-20) (n = 14)**	**Cromolyn 40 mg (SCG-40) (n = 12)**	**p-value **
**FEV1 **	Baseline	60.07 ± 3.29	57.33 ± 3.54	67.49 ±2.04	NS
	15 min	60.56 ± 3.29	58.85 ± 3.22	66.97 ± 1.99	NS
	30 min	60.46 ± 3.27	58.41 ± 2.90	67.29 ± 2.00	NS
	60 min	62.06 ± 3.17	59.49 ± 3.40	67.97 ± 2.04	NS
**FVC **	Baseline	71.17 ± 3.30	70.17 ± 3.23	73.45 ±7.40	NS
	15 min	71.71 ± 3.37	71.30 ± 3.23	73.75 ±2.52	NS
	30 min	71.94 ± 3.24	71.22 ±2.63	73.02 ± 2.72	NS
	60 min	72.77 ± 3.76	72.53 ± 2.85	74.33 ± 2.61	NS
**PEF **	Baseline	59.19 ±4.91	56.02 ± 4.62	64.79 ± 2.71	NS
	15 min	57.85 ± 4.68	58.93 ± 4.48	65.10 ±3.60	NS
	30 min	58.66 ± 5.68	58.74 ± 4.51	65.50 ±3.40	NS
	60 min	58.56 ± 5.23	60.69 ± 4.76	65.20 ± 3.13	NS
**MEF75% **	Baseline	41.52 ±5.45	38.86 ± 4.41	54.37 ± 3.21*	S
	15 min	41.87 ± 4.56	39.73 ± 4.47	52.93 ± 3.38	-
	30 min	42.81 ± 3.88	38.69 ±3.94	52.59 ± 3.55	-
	60 min	44.31 ± 3.80	38.37 ± 4.17	53.91 ± 3.49	-
**MEF50% **	Baseline	34.87 ± 4.89	29.96 ± 3.75	45.62 ± 3.25*	S
	15 min	33.73 ± 3.79	31.67 ± 3.68	44.30 ± 2.49	-
	30 min	32.78 ± 2.82	30.30 ± 3.41	44.97 ± 2.58	-
	60 min	36.64 ±3.18	29.91 ± 3.13	45.68 ± 2.63	-
**MEF25% **	Baseline	30.78 ± 4.91	24.76 ± 2.64	39.76 ± 2.24*	S
	15 min	29.79 ± 4.63	26.89 ± 3.31	39.80 ± 2.69	-
	30 min	27.48 ± 3.48	26.25 ± 2.76	42.24 ±2.50	-
	60 min	30.66 ± 3.95	25.22 ± 2.56	39.56 ± 2.62	-

SCG-P: Sodium cromoglycate-placebo group, SCG-20: Sodium cromoglycate 20 mg group, SCG-40: Sodium cromoglycate 40 mg group, -: Weren’t compared, NS: Non-significant, S: Significant, *p< 0.05 compared with related value in SCG-20

**Figure 1:**
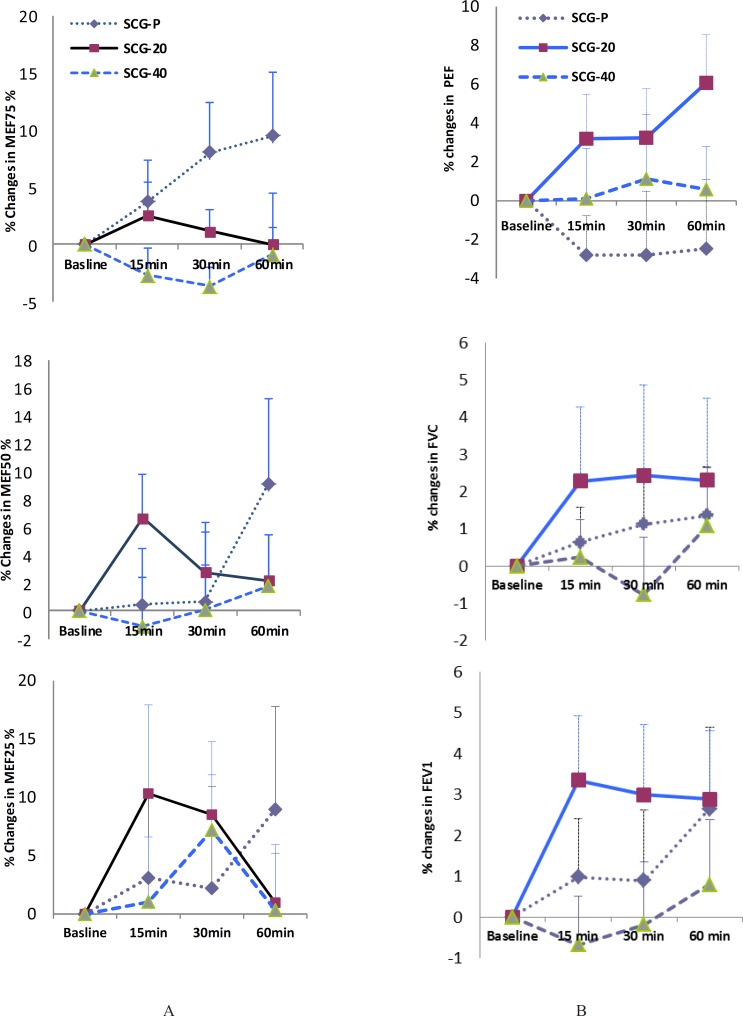
(A) Effects of a single dose of sodium cromoglycate (cromolyn) on maximal expiratory flow when 75%, 50% and 25% of the forced vital capacity remained in the lungs, which are MEF75%, MEF50% and MEF25% respectively, in adult asthmatics. SCG-P: placebo group, SCG-20: cromolyn 20 mg group, SCG-40: cromolyn 40 mg group. (B) Effects of a single dose of sodium cromoglycate (cromolyn) on lung function variables in adult asthmatics. PEF: peak expiratory flow, FEV1: Forced expiratory flow in one second, FVC: forced vital capacity. SCG-P: placebo group, SCG-20: cromolyn 20 mg group, SCG-40: cromolyn 40 mg group. Data are expressed as mean ± SEM. n = 12 in cromolyn 40 mg, n = 14 in placebo and cromolyn 20 mg

Patients in SCG-20 have had greater improvement in their PEF, FEV1 and FVC at 15 min, compared with those in other groups. Similar improvements also were observed for MEF50% and MEF25% at same time (SCG-20 vs. SCG-P). The positive effects of 20 mg cromolyn continued for FEV1, FVC, and PEF, but didn’t continue for the small caliber airways MEF50% and MEF25%, at 30 min and 60 min. However, for all parameters the differences between means of change from baseline value of those tests were not statistically significant (Figure 1).

Patients were observed for an hour after the last test. No significant adverse effect or asthma attack was developed after the inhalation of drug or placebo. Some patients in cromolyn groups had complaints of headache, throat burning, bitter taste or developed cough that resolved in a few minutes.

The present work is the first study evaluating the effect of short course treatment (as a single dose) with cromolyn in Iranian asthmatics, which weren’t subjected to any known challenge. This study was a double-blind, randomized placebo controlled clinical trial. We found that asthmatic patients which inhaled 20 mg cromolyn sodium had more improvement in most of their lung function variables compared with those of asthmatics inhaled 40 mg cromolyn sodium or cromolyn-placebo. This improvement occurred for the FEV_1_, FVC, PEF (during study), and for the small caliber airways MEF_50%_ and MEF_25%_ (Figure 1). This relatively better response occurred in conditions which patients in SCG-20 group have a relatively more severe asthma. This is evident by their relatively lower baseline FEV_1_ compared to those of SCG-P and SCG-40 (57.3% *vs*. 60% and 67%, respectively, as indicated in Table 1). In contrast, the dosage of 40 mg of cromolyn has relatively negative effects on lung function. The latter may be due to the irritant effect of many powder particles of inhaled SCG at this high dosage on the inflamed airways. However, these changes weren’t statistically significant. Findings of our work are supported via the previous studies (16, 21, 22). Tullett *et al.* studied the effects of 2, 10 and 20 mg of SCG delivered via aerosol on exercise-induced asthma. The FEV_1_ was recorded before the treatment, 30 min after the treatment before the exercise, and up to 30 min after the exercise. They reported that mean baseline values of FEV_1_, before and after the placebo or SCG did not differ significantly (21). In addition, in other investigation, the protective effects of inhaled SCG in increasing the concentration from 2 to 40 g/L were evaluated in exercise-induced asthma. The FEV_1_ was recorded before and 20 min after the inhalation of saline (as control) and SCG, also up to 30 min after the exercise testing on 4 days. There was no significant difference between the mean baseline values of FEV_1_ before and after the saline and SCG during the study (22). In other study which evaluated the protective effect of terbutaline sulfate and cromolyn sodium in exercise-induced asthma, it was reported that there was no significant change in FEV_1_, 10 min after cromolyn sodium or placebo (16). In these studies, the effect of cromolyn is studied up to 30 min of the inhalation, but in present work, the time course of the effect of cromolyn is studied for 60 min.

Individual group analysis (data are not shown) showed that unlike other groups, low dose of cromolyn induced a significant bronchodilation in two patients (13.5% of patients) 15 min after using the drug. At this time, the average improvement of FEV_1_ from the baseline was 14.5%. At other time points, also two patients (13.5% of patients) in each of SCG-20 or SCG-P groups have such response. The averages of FEV_1_ improvement percentage were 13 and 14.2, at 30 and 60 min respectively, in SCG-P group and 15.3 and 13.45 at 30 and 60 min respectively, in SCG-20 group. This finding is according to the criteria of American Thoracic Society (ATS) for a «significant response» in adults: 12% improvement from the baseline value and a 0.2 L increase in either FEV_1_ or FVC (18, 23). Therefore, in the present study, significant bronchodilation developed in a few patients in both SCG-P and SCG-20 groups, but not in SCG-40 group. However, unlike the SCG-P, the «significant response» for SCG-20 developed 15 min sooner and was associated with similar improvement in related FVC values. Bronchodilation (bronchial responsiveness) is an integrated physiologic mechanism involving airway epithelium, nerve, mediators and bronchial smooth muscle (23). In asthmatic patients, bronchodilation may develop spontaneously or due to the drug (18). The development of a significant response to low dose of cromolyn in a few patients in our trial could be explained through some studies which showed that cromolyn can modulate the airway smooth muscle function *in-vitro *(24, 25). Kitamura *et al.* investigated the effect of SCG on the action of various bronchoactive agents in isolated guinea-pig tracheal strips. SCG attenuated the acetylcholine-induced contractile responses and shifted the dose-response curve of acetylcholine downward. They suggested that SCG might have a direct action on bronchial smooth muscle in addition to the inhibition of chemical mediators release from the mast cells (24).

## Conclusions

Sodium cromolyn and nedocromil are members of a group of effective anti-asthma drugs which are only effective in preventing asthma attack. Besides, they have protective effect against the exercise-induced asthma. Results of the present work showed that 20 mg of SCG improved the lung function slightly more than the other groups in non-smoker stable asthmatics, while the difference between the means of changes was not statistically significant. As well, unlike the other groups, a few patients inhaling 20 mg cromolyn, undergo a level of improvement in their FEV_1_ at the first time (15 min), which was defined as the criteria of «significant response to brochodilator» (ATS). This may reflect the especial response of some (but not all) asthmatic subjects to cromolyn sodium. The importance of this finding in the final effectiveness of drug in these patients is not clear. The clarification of this finding needs a larger and more detailed study in this field.
